# Supplementary material for: New Insights on Glutathione’s Supramolecular Arrangement and Its In Silico Analysis as an Angiotensin-Converting Enzyme Inhibitor
Source: Molecules. 2022 Nov 17;27(22):7958. doi: 10.3390/molecules27227958 (PMC9695799; doi:10.3390/molecules27227958)
Supplement: Supplementary file 1 [file molecules-27-07958-s001.zip › molecules-1968684-supplementary.pdf]

New insights on Glutathione's Supramolecular Arrangement and Its In Silico Analysis as an Angiotensin-Converting Enzyme Inhibitor

Antônio S. N. Aguiar [1,*], Igor D. Borges [1,2], Leonardo L. Borges [1,3], Lucas D. Dias [1,4], Ademir J. Camargo [1], Pál Perjesi [4] and Hamilton B. Napolitano [1,4,*]

[1] Grupo de Química Teórica e Estrutural de Anápolis, Universidade Estadual de Goiás, Anápolis, GO, Brazil
[2] Centro de Pesquisa e Eficiência Energética, CAOA Montadora de Veículos LTDA, Anápolis, GO, Brazil
[3] Escola de Ciências Médicas e da Vida, Pontifícia Universidade Católica de Goiás, Goiânia, GO, Brazil
[4] Laboratório de Novos Materiais, Universidade Evangélica de Goiás, Anápolis 75083-515, GO, Brazil
* Correspondence: toninho.quimica@gmail.com (A.S.N.A.); hbnapolitano@gmail.com (H.B.N.)

SUPPLEMENTARY MATERIAL

Table S1. Second-order perturbation theory analysis in NBO basis obtained at M06-2X/6-311++G(d,p) level of theory for the GSHA polymorph.

Interaction	Occupancy	Donor NBO (i)	Hybrids	Occupancy	Acceptor NBO (j)	Hybrids	$E^{(2)}$ (kcal/mol)	$\varepsilon_{(j)}$- $\varepsilon_{(i)}$ (a.u.)	$F(i,j)$ (a.u.)
S–H$\cdots$$O_2$	1.99199	η1(S)	S: $sp^{0.66}$	0.06022	σ*(C_5-O_2)	C_5: $sp^{2.04}$<br>O_2: $sp^{1.80}$	0.07	1.27	0.009
	1.99341	σ(C_5-O_2)	C_5: $sp^{2.04}$<br>O_2: $sp^{1.80}$	0.01311	σ*(S-H)	S: $sp^{3.34}$<br>H: s	0.08	1.47	0.01
	1.98348	π(C_5-O_2)	C_5: p<br>O_2: p	0.01311	σ*(S-H)	S: $sp^{3.34}$<br>H: s	0.98	0.84	0.026
	1.88395	$η_2$(O_1)	O_1: p	0.01311	σ*(S-H)	S: $sp^{3.34}$<br>H: s	0.10	0.74	0.008
	1.9791	$η_1$(O_2)	O_2: $sp^{0.58}$	0.01311	σ*(S-H)	S: $sp^{3.34}$<br>H: s	0.24	1.2	0.015
	1.88406	$η_2$(O_2)	O_2: p	0.01311	σ*(S-H)	S: $sp^{3.34}$<br>H: s	1.53	0.72	0.03
N_2–H$\cdots$$O_2$	1.71526	$η_1$(N_2)	N_2: p	0.05091	σ*(C_5-O_5)	C_5: $sp^{1.94}$<br>O_2: $sp^{1.68}$	0.24	0.91	0.014
	1.97429	$η_1$(O_2)	O_2: $sp^{0.60}$	0.0208	σ*(N_2-H)	N_2: $sp^{1.95}$<br>H: $sp^{1.68}$	2.01	1.69	0.052
	1.89151	$η_2$(O_2)	O_2: p	0.0208	σ*(N_2-H)	N_2: $sp^{1.95}$<br>H: $sp^{1.68}$	2.93	1.21	0.054
O_6–H$\cdots$$O_3$	1.98034	σ(O_6-H)	O_6: $sp^{2.01}$<br>H: $sp^{2.01}$	0.02804	σ*(C_4-O_3)	C_4: $sp^{2.20}$<br>O_3: $sp^{1.49}$	0.13	1.59	0.013
	1.98034	σ(O_6-H)	O_6: $sp^{2.01}$<br>H: $sp^{2.01}$	0.29654	π*(C_4-O_3)	C_4: p<br>O_3: p	0.20	1.01	0.014
	1.95731	$η_1$(O_6)	O_6: $sp^{2.89}$	0.02804	σ*(C_4-O_3)	C_4: $sp^{2.20}$<br>O_3: $sp^{1.49}$	0.13	1.17	0.011
	1.95731	$η_1$(O_6)	O_6: $sp^{2.89}$	0.07635	π*(C_8-O_4)	C_8: $sp^{2.80}$<br>O_4: $sp^{2.26}$	1.83	0.75	0.035
	1.96552	$η_1$(O_3)	O_3: $sp^{0.73}$	0.03126	σ*(O6-H)	O_6: $sp^{2.01}$<br>H: s	8.52	1.71	0.108

	1.88081	$\eta_2(O_3)$	O_3:	*p*	0.03126	σ*(O6-H)	O_6:<br>H:	$sp^{2.01}$<br>*s*	9.45	1.27	0.100
	1.9927	$\sigma(C_4\text{-}O_3)$	C_4<br>O_3	$sp^{2.20}$<br>$sp^{1.49}$	0.03126	σ*(O6-H)	O_6:<br>H:	$sp^{2.01}$<br>*s*	0.16	2.07	0.016
	1.98922	$\pi(C_4\text{-}O_3)$	C_4:<br>O_3:	*p*<br>*p*	0.03126	σ*(O6-H)	O_6:<br>H	$sp^{2.01}$<br>*s*	1.50	1.4	0.041
N_3–H···O_5	1.68454	η_1(N3)	N_3:	*p*	0.02682	$\sigma^*(C_{10}\text{-}O_5)$	C_{10}:<br>O_5:	$sp^{1.94}$<br>$sp^{1.42}$	0.14	1	0.012
	1.86223	$\eta_2(O_5)$	O_5:	*p*	0.01319	σ*(N3-H)	N_3:<br>H:	$sp^{2.12}$<br>*s*	1.63	1.21	0.041
C_2–H···O_4	1.97281	$\sigma(C_2\text{-}H)$	C_2:<br>H:	$sp^{2.74}$<br>*s*	0.0208	σ*(N2-H)	N_2:<br>H:	$sp^{1.95}$<br>*s*	0.27	1.56	0.018

Table S2. Second-order perturbation theory analysis in NBO basis obtained at M06-2X/6-311++G(d,p) level of theory for the GSHB polymorph.

Interaction	Occupancy	Donor NBO (i)	Hybrids	Occupancy	Acceptor NBO (j)	Hybrids	$E^{(2)}$ (kcal/mol)	$\varepsilon_{(j)}$- $\varepsilon_{(i)}$ (a.u.)	$F(i,j)$ (a.u.)
S–H···O$_1$	1.96681	σ(C$_9$-H)	C$_9$: $sp^{3.03}$<br>H: s	0.01589	σ*(C$_2$-H)	C$_2$: $sp^{3.50}$<br>H: s	0.72	1.04	0.025
	1.99282	η$_1$(S)	S: $sp^{0.50}$	0.05303	σ*(O$_1$-C$_5$)	O$_1$: $sp^{1.57}$<br>C$_5$: $sp^{1.98}$	0.07	1.37	0.009
	1.97472	σ(C$_2$-H)	C$_2$: $sp^{3.50}$<br>H: s	0.01790	σ*(C$_9$-H)	C$_9$: $sp^{3.03}$<br>H: s	0.94	1.05	0.028
	1.97342	η$_1$(O$_1$)	O$_1$: $sp^{0.64}$	0.01938	σ*(S-H)	S: $sp^{4.87}$<br>H: s	2.23	1.04	0.043
	1.87540	η$_2$(O$_1$)	O$_1$: p	0.01938	σ*(S-H)	S: $sp^{4.87}$<br>H: s	0.61	0.57	0.017
	1.62112	η$_3$(O$_1$)	O$_1$: p	0.01938	σ*(S-H)	S: $sp^{4.87}$<br>H: s	2.56	0.54	0.037
N$_1$–H···O$_1$	1.98990	σ(N$_1$-H)	N$_1$: $sp^{2.87}$	0.05358	σ*(O$_1$-C$_5$)	O$_1$: $sp^{1.54}$<br>C$_5$: $sp^{1.97}$	0.10	1.41	0.011
	1.99493	σ(O$_1$-C$_5$)	O$_1$: $sp^{1.54}$<br>C$_5$: $sp^{1.97}$	0.04675	σ*(N$_1$-H)	N$_1$: $sp^{3.10}$<br>H: s	0.05	1.58	0.008
	1.96840	η$_1$(O$_1$)	O$_1$: $sp^{0.65}$	0.04675	σ*(N$_1$-H)	N$_1$: $sp^{3.10}$<br>H: s	6.25	1.26	0.079
	1.87638	η$_2$(O$_1$)	O$_1$: p	0.04675	σ*(N$_1$-H)	N$_1$: $sp^{3.10}$<br>H: s	1.73	0.79	0.034
	1.62453	η$_3$(O$_1$)	O$_1$: p	0.04675	σ*(N$_1$-H)	N$_1$: $sp^{3.10}$<br>H: s	0.95	0.76	0.026
	1.99091	σ(N$_1$-H)	N$_1$: $sp^{2.73}$	0.05358	σ*(O$_1$-C$_5$)	O$_1$: $sp^{1.54}$<br>C$_5$: $sp^{1.97}$	0.09	1.41	0.01
N$_1$–H···O$_2$	1.99086	σ(N$_1$-H)	N$_1$: $sp^{3.10}$	0.04917	σ*(O$_2$-C$_5$)	O$_1$: $sp^{1.53}$<br>C$_5$: $sp^{2.01}$	0.11	1.29	0.011
	1.99086	σ(N$_1$-H)	N$_1$: $sp^{3.10}$	0.37882	π*(O$_2$-C$_5$)	O$_1$: p<br>C$_5$: p	0.10	0.72	0.008
	1.99255	σ(O$_2$-C$_5$)	O$_2$: $sp^{1.73}$<br>C$_5$: $sp^{1.95}$	0.03759	σ*(N$_1$-H)	N$_1$: $sp^{2.73}$<br>H: s	0.24	1.61	0.018
	1.87311	η$_2$(O$_2$)	O$_2$: p	0.03759	σ*(N$_1$-H)	N$_1$: $sp^{2.73}$<br>H: s	15.51	0.87	0.106
N$_2$–H···O$_2$	1.98365	σ(N$_2$-H)	N$_2$: $sp^{2.34}$<br>H:	0.37882	π*(O$_2$-C$_5$)	O$_2$: p<br>C$_5$: p	0.18	1.3	0.014
	1.99255	σ(O$_2$-C$_5$)	O$_2$: $sp^{1.73}$<br>C$_5$: $sp^{1.95}$	0.03353	σ*(N$_2$-H)	N$_2$: $sp^{2.34}$<br>H: s	0.15	1.65	0.014
	1.95852	η$_2$(O$_2$)	O$_2$: p	0.03353	σ*(N$_2$-H)	N$_2$: $sp^{2.34}$<br>H: s	6.80	1.36	0.086
C$_3$–H···O$_2$	1.97934	σ(C$_3$-H)	C$_3$: $sp^{3.41}$<br>H: s	0.37882	σ*(O$_2$-C$_5$)	O$_2$: p<br>C$_5$: p	0.12	0.55	0.008
	1.68940	η$_1$(N$_3$)	N$_3$: p	0.02517	σ*(C$_6$-H)	C$_6$: $sp^{3.22}$<br>H: s	0.06	0.71	0.006
N$_1$–H···O$_4$	1.99182	σ(N$_1$-H)	N$_1$: $sp^{2.85}$	0.06532	σ*(O$_4$-C$_8$)	O$_4$: $sp^{1.94}$	0.17	1.3	0.013

			H:	$sp^{2.85}$			C_8:	$sp^{2.73}$			
	1.98896	$\pi(O_4\text{-}C_8)$	O_4:	p	0.03821	$\sigma^*(N_1\text{-}H)$	N_1:	$sp^{2.85}$	0.06	1.02	0.007
			C_8:	p			H:	s			
	1.96655	$\eta_1(O_4)$	O_4:	$sp^{0.72}$	0.03821	$\sigma^*(N_1\text{-}H)$	N_1:	$sp^{2.85}$	6.64	1.25	0.082
							H:	s			
	1.88004	$\eta_2(O_4)$	O_4:	p	0.03821	$\sigma^*(N_1\text{-}H)$	N_1:	$sp^{2.85}$	9.61	0.82	0.081
							H:	s			
$N_3\text{–}H\cdots O_5$	1.98257	$\sigma(N_3\text{-}H)$	N_3:	$sp^{2.37}$	0.03602	$\sigma^*(O_5\text{-}C_{10})$	O_5:	$sp^{1.62}$	0.10	1.35	0.011
			H:	s			C_{10}:	$sp^{2.13}$			
	1.68499	$\eta_1(N_3)$	N_3:	p	0.03602	$\sigma^*(O_5\text{-}C_{10})$	O_5:	$sp^{1.62}$	0.05	0.91	0.007
							C_{10}:	$sp^{2.13}$			
	1.68499	$\eta_1(N_3)$	N_3:	p	0.20915	$\pi^*(O_5\text{-}C_{10})$	O_5:	p	0.05	0.36	0.004
							C_{10}:	p			
	1.96926	$\eta_1(O_5)$	O_5:	$sp^{0.69}$	0.05430	$\sigma^*(O_3\text{-}C_4)$	O_3:	$sp^{1.87}$	0.14	1.41	0.013
							C_4:	$sp^{2.51}$			
	1.96926	$\eta_1(O_5)$	O_5:	$sp^{0.69}$	0.22712	$\pi^*(O_3\text{-}C_4)$	O_3:	p	1.16	0.97	0.032
							C_4:	p			
	1.96926	$\eta_1(O_5)$	O_5:	$sp^{0.69}$	0.03034	$\sigma^*(N_3\text{-}H)$	N_3:	$sp^{2.37}$	4.29	1.34	0.068
							H:	s			
	1.86870	$\eta_2(O_5)$	O_5:	p	0.03034	$\sigma^*(N_3\text{-}H)$	N_3:	$sp^{2.37}$	3.07	0.89	0.048
							H:	s			
$O_6\text{–}H\cdots O_3$	1.98322	$\sigma(O_6\text{-}H)$	O_6:	$sp^{2.67}$	0.21210	$\pi^*(O_3\text{-}C_4)$	O_3:	p	0.24	1.03	0.015
			H:	s			C_4:	p			
	1.99014	$\sigma(O_3\text{-}C_4)$	O_3:	$sp^{2.41}$	0.05759	$\sigma^*(O_6\text{-}H)$	O_6:	$sp^{2.67}$	0.46	1.53	0.024
			C_4:	$sp^{3.18}$			H:	s			
	1.98680	$\pi(O_3\text{-}C_4)$	O_3:	p	0.05759	$\sigma^*(O_6\text{-}H)$	O_6:	$sp^{2.67}$	0.18	1.18	0.013
			C_4:	p			H:	s			
	1.96252	$\eta_1(O_3)$	O_3:	$sp^{0.74}$	0.05759	$\sigma^*(O_6\text{-}H)$	O_6:	$sp^{2.67}$	7.80	1.33	0.092
							H:	s			
	1.86499	$\eta_2(O_3)$	O_3:	p	0.05759	$\sigma^*(O_6\text{-}H)$	O_6:	$sp^{2.67}$	22.01	0.93	0.13
							H:	s			
